# Correction: Correlative live and super-resolution imaging reveals the dynamic structure of replication domains

**DOI:** 10.1083/JCB.20170907408082018c

**Published:** 2018-09-03

**Authors:** Wanqing Xiang, M. Julia Roberti, Jean-Karim Hériché, Sébastien Huet, Stephanie Alexander, Jan Ellenberg

Vol. 217, No. 6, June 4, 2018. 10.1083/jcb.201709074.

The authors noticed the erroneous duplication of an image between the 60- and 120-min time points in the top row of Fig. 3 B. The correct images for the 120-min time point have been added (first row, full-size and magnification). To clarify the presentation in the first-row magnifications (time points 30 min, 60 min, 90 min, and 120 min), the authors also replaced the previously found arrows with ellipses to indicate exemplary pairs between which distances were measured. The figure legend has been modified accordingly.

**Figure 3.**
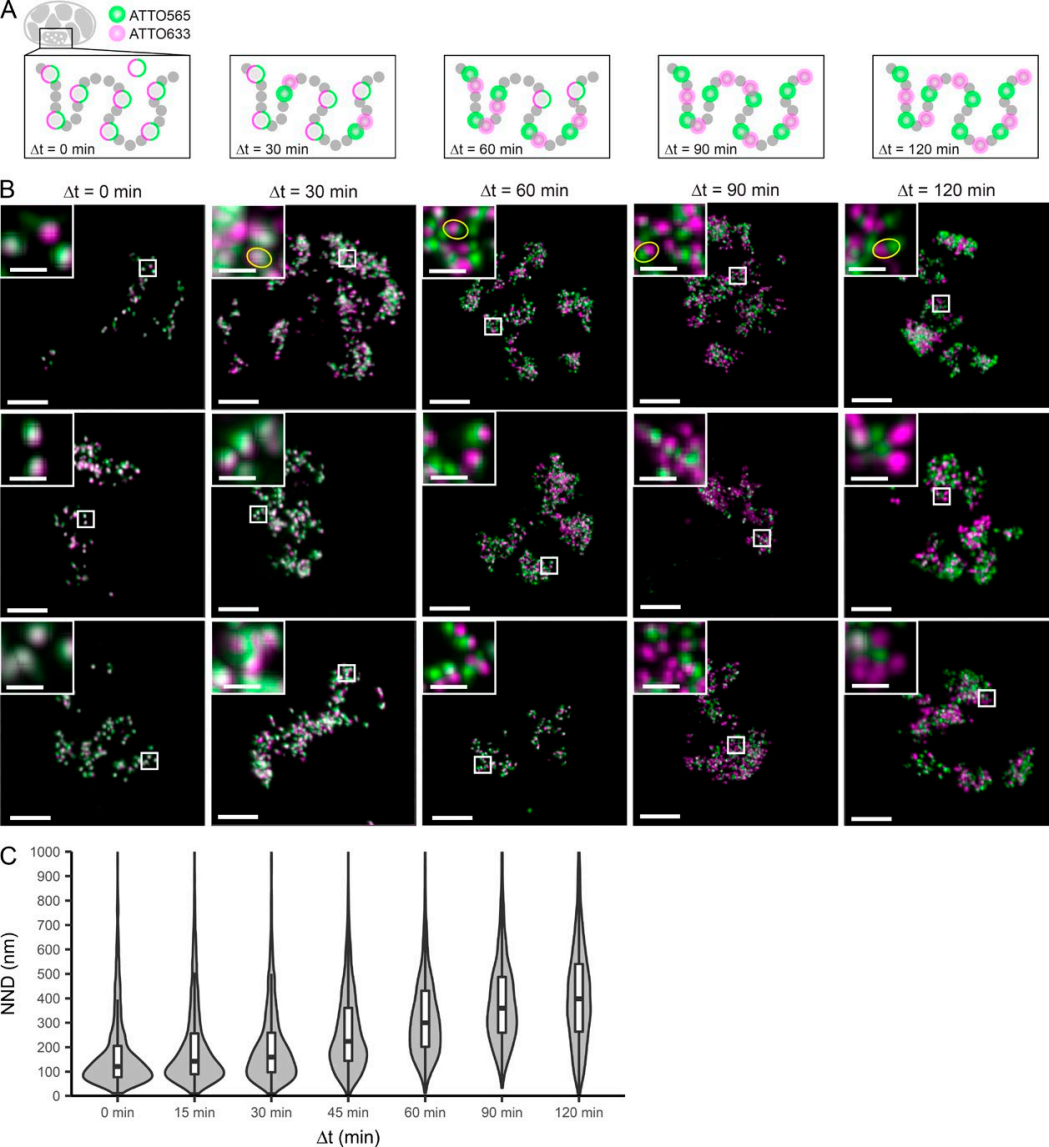
**Dual-color confocal imaging shows neighboring domains spacing. (A)** Schematics of the labeling pattern progression. A first round of co-replicative labeling with ATTO 633-dUTP yields a first set of labeled RDs (green circles). A second labeling round with ATTO 565-dUTP is performed at increasing Δt targets with either the same RDs or first/second neighboring RDs, depending on Δt (magenta circles). The typical interval required to complete replication of one RD and proceed to the neighboring one is Δt = 60 min (Jackson and Pombo, 1998); therefore, we applied Δt = 0, 15, 30, 45, 60, 90, and 120 min (for the sake of space, Δt = 15 and 45 are not depicted). **(B)** Images showing cells subjected to double-color labeling at Δt = 0, 30, 60, 90, and 120 min. Images correspond to maximum-intensity projected z-stacks after deconvolution (50-nm pixels in x,y, 150-nm pixels in z) and overlay of ATTO 633 (green) and ATTO 565 (magenta) channels. Bars: 5 µm; (insets) 1 µm. Small boxes mark the position of the insets. Insets show zoomed-in detailed view of dual-color–labeled RDs, with yellow ellipses indicating exemplary pairs between which distances were measured and used to estimate median NND of neighboring RDs. **(C)** Violin plots showing the distribution and median NND between pairs of ATTO 633- and ATTO 565-dUTP–labeled RDs at increasing Δt. Δt = 0 min (*n* = 1,170 pairs, 11 cells), 15 min (*n* = 3,090 pairs, 15 cells), 30 min (*n* = 2,677 pairs, 15 cells), 45 min (*n* = 4,097 pairs, 15 cells), 60 min (*n* = 2,711 pairs, 14 cells), 90 min (*n* = 2,367 pairs, 16 cells), and 120 min (*n* = 2,939 pairs, 15 cells). Distances >1,000 nm are not shown but are included in the determination of the quantiles. The median NND at Δt = 60 min was used as estimation on the nearest neighboring RD spacing.

Both the HTML and PDF versions of the article have been corrected. These errors appear only in print and PDF versions downloaded on or before August 21, 2018.

