# Supplementary material for: Correlative live and super-resolution imaging reveals the dynamic structure of replication domains
Source: J Cell Biol. 2018 Jun 4;217(6):1973–84. doi: 10.1083/jcb.201709074 (PMC5987722; doi:10.1083/jcb.201709074)
Supplement: Supplemental Materials (PDF) [file JCB_201709074_sm.pdf]

Supplemental material

Xiang et al., https://doi.org/10.1083/jcb.201709074

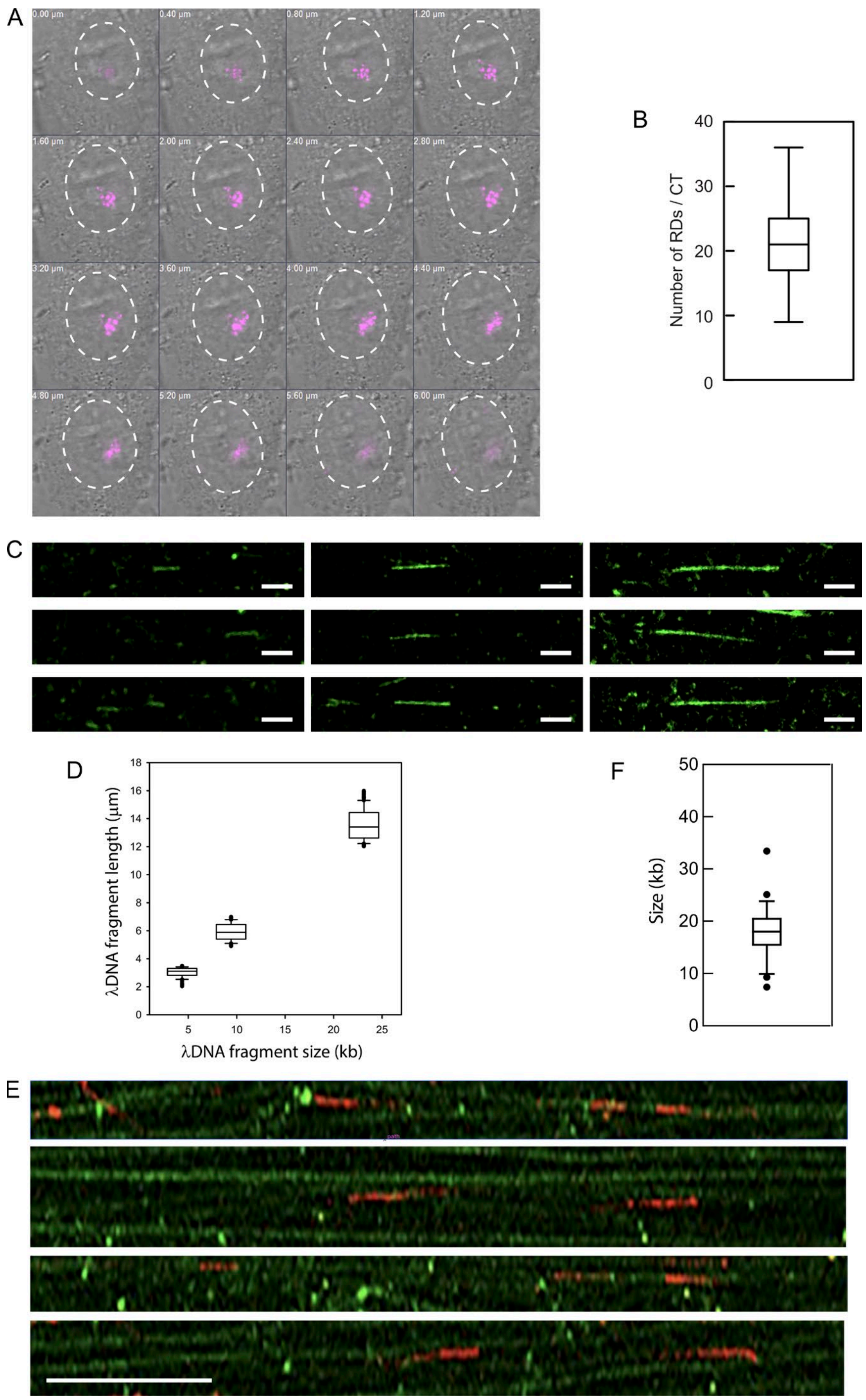

Figure S1. **Characterization of co-replicative labeling of early-replicating RDs for correlative confocal-STORM imaging. (A)** Example of an NRK with a single CT labeled with ATTO 633-dUTP (magenta). The transmission image, along with an outline of the nuclear envelope is shown (gray image and dashed white line). The spatial distribution of RDs within the nuclear volume was captured with a z-stack. **(B)** Estimation of the number of RDs/CT (n = 71 cells). **(C)** Examples of λDNA fragments used in DNA-combing experiments to calibrate the extent of DNA stretching as a function of DNA kilobase. Left panels: 4.36 kb; middle panels: 9.41 kb; right panels: 23.13 kb. Bar, 5 µm. **(D)** Calibration of the DNA length as a function of DNA size in kilobase, from measurements performed on stretched λDNA fragments (sizes as described in C). **(E)** DNA-combing performed on DNA content from NRKs subjected to replicative labeling with ATTO 633-dUTP (magenta). The DNA fiber was counterstained with PicoGreen (green). Bar, 30 µm. **(F)** Size of co-replicating regions estimated from the size of the ATTO 633-dUTP–labeled DNA backbone stretch.

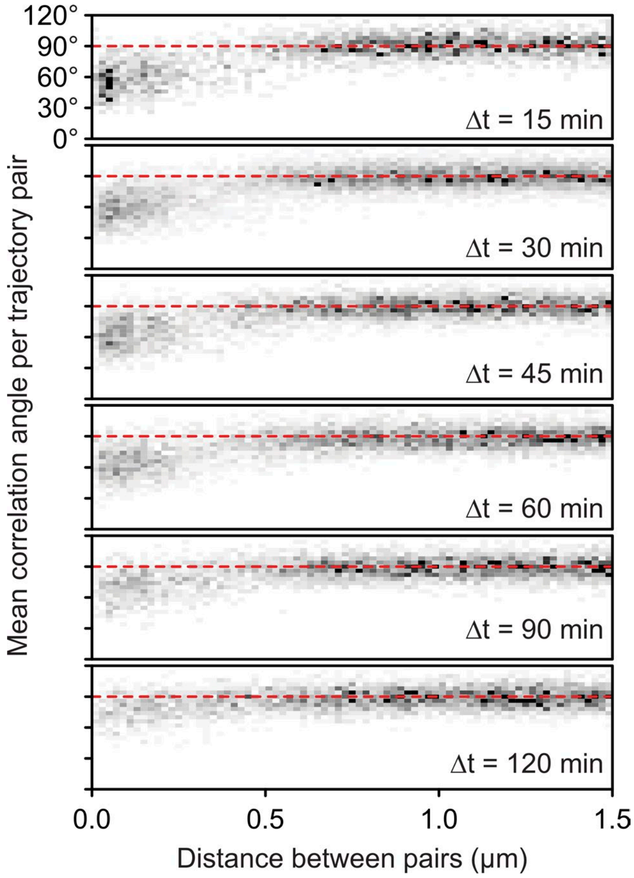

Figure S2. **Dual-color live-cell confocal imaging measures a weak mechanical coupling of neighboring RDs.** Averaged mean correlation angle <α> is extracted as a function of distance between pairs of RDs labeled with ATTO 633 and ATTO 565. The individual plots show the angles corresponding to unpooled data from Δt = 15 min (*n* = 3,144 pairs, 26 cells), 30 min (*n* = 8,256 pairs, 27 cells), 45 min (*n* = 5,126 pairs, 21 cells), 60 min (*n* = 5,306 pairs, 15 cells), 90 min (*n* = 4,103 pairs, 22 cells), and 120 min (*n* = 4,329 pairs, 22 cells).

Table S1. **Fluorophores tested for optimized replicative labeling of RDs by using aminoallyl-dUTP derivatives in correlative confocal super-resolution imaging applications**

Fluorophore-dUTP 5-(3-aminoallyl) derivative	Single color		Dual color, confocal
	Confocal	STORM	
ATTO 532	++	++	++
Abberior CAGE 532	++	-	++
Abberior CAGE 552	++	+	++
Cy3	+++	+	+++
Cy3B	++	+	+++
CF568	+++	+	+++
TMR	+++	+	+++
ATTO 565	+++	+	+++
ATTO 633	+++	+++	+++
Alexa Fluor 647	+	+++	+
DyLight677	+++	+	+++
Alexa Fluor 680	–	++	–
CF 680	–	–	–

The performance was qualitatively assessed by visual inspection of confocal/STORM images of the fluorophores, and ranked as excellent (+++), very good (++), acceptable (+), or not suitable (–).
